# High-Order Wave-Damage Interaction Coefficients (WDIC) Extracted through Modal Decomposition

**DOI:** 10.3390/s21082749

**Published:** 2021-04-13

**Authors:** Hanfei Mei, Victor Giurgiutiu

**Affiliations:** Department of Mechanical Engineering, University of South Carolina, 300 Main Street, Columbia, SC 29208, USA; hmei@email.sc.edu

**Keywords:** guided waves, wave damage interaction, high-order modes, modal decomposition, finite element modeling, scattering, mode conversion

## Abstract

This paper presents a new technique for the extraction of high-order wave-damage interaction coefficients (WDIC) through modal decomposition. The frequency and direction dependent complex-valued WDIC are used to model the scattering and mode conversion phenomena of guided wave interaction with damage. These coefficients are extracted from the harmonic analysis of local finite element model (FEM) mesh with non-reflective boundaries (NRB) and they are capable of describing the amplitude and phase of the scattered waves as a function of frequency and direction. To extract the WDIC of each wave mode, all the possible propagating wave modes are considered to be scattered simultaneously from the damage and propagate independently. Formulated in frequency domain, the proposed method is highly efficient, providing an overdetermined equation system for the calculation of mode participation factors, i.e., WDIC of each mode. Case studies in a 6-mm aluminum plate were carried out to validate the WDIC of: (1) a through-thickness hole and (2) a sub-surface crack. At higher frequency, scattered waves of high-order modes will appear and their WDIC can be successfully extracted through the modal decomposition.

## 1. Introduction

Guided waves can propagate over a long distance in thin-plate structures, which retains a crucial role in the development of structural health monitoring (SHM) and nondestructive evaluation (NDE) systems using piezoelectric wafer active sensors (PWAS) [1,2,3,4,5,6,7]. The development of computational models for guided wave interaction with damage is important to the SHM system design and sensing signal interpretation [8]. However, the modeling of wave damage interaction is challenging because it involves complicated phenomena such as wave transmission, reflection, mode conversion and generation of nonlinear higher harmonics.

Regarding the aspect of wave-damage interaction, many researchers have developed analytical models using Kirchhoff, Mindlin, Kane-Mindlin plate theory and three-dimensional (3D) elasticity solution or exact Lamb mode solutions [9,10,11,12,13,14,15,16]. The scattering at a through-thickness hole was calculated analytically and compared to experiments for fundamental A0 mode [10] and S0 mode [11]. Moreover, the scattering and mode conversion were studied analytically at cylindrical inhomogeneity [14] and part-thickness circular holes [15]. The advantage of analytical models is that they are fast, efficient and capable of providing parametric studies, but the drawback is that they only apply to simple damage geometries such as through-thickness holes [9,10,11], partial-through-thickness holes [13,15], or flat-bottom cavities [16] in isotropic materials.

To model the wave interaction with damage of complex geometries, many researchers have put emphasis on finite element modeling (FEM). In recent years, the development of FEM techniques allows us to study conveniently the scattering and mode conversion phenomena in bulk waves and guided waves from complicated damage [17]. FEM is becoming a popular tool for understanding the complex problems of wave damage interaction. The scattering of guided waves from various types of damage has been analyzed by using FEM simulations. Fromme [18] investigated the scattering of A0 mode at through-thickness and part-through crack-like defects through FEM and detailed experimental measurements. Veidt and Ng [19] applied transient FEM analysis to study guided wave scattering due to through-thickness holes in unidirectional, cross-ply and quasi-isotropic laminates. They investigated the influence of the stacking sequence. The scattering at delamination in composite plates was also implemented [20,21]. Mural et al. [22] used FEM transient analysis to obtain the scattered field around the impact-induced delamination and they investigated the effect of delamination size and shape. However, these time-domain FEM simulations have to be re-run for each test frequency. To overcome this problem, Shen and Giurgiutiu [23] proposed a hybrid global-local (HGL) approach for the accurate and efficient simulation of guided wave propagation and interaction with damage in isotropic plates. A global analytical solution was used to model wave generation and propagation, while the wave-damage interaction coefficients (WDIC) were extracted from the harmonic analysis of local FEM in the damaged regions to describe the wave damage interaction. The harmonic analysis of local FEM is faster and more target-oriented compared with conventional time transient analysis. Besides, the harmonic analysis does not require the results of the previous calculation step to solve the current step, as is the case in conventional transient analysis. Bhuiyan et al. [24] extended the harmonic analysis of local FEM to obtain the WDIC scatter cubes, which can describe the 3D interaction (frequency, incident direction and azimuth direction) of guided waves with the damage. However, the extraction methodology considered in [23,24] was designed especially for only fundamental guided wave modes (S0, A0 and SHS0).

It is known that high-order modes will appear at a high frequency-thickness regime. Aside from the fundamental modes, high-order modes (A1, A2, S1 and S2) were also generated in a 6.35-mm-thick steel plate using broadband excitation [25]. High-order modes have been used for material characterization through the measured dispersion curves with the advantage of making a precise inversion of the model parameters to evaluate material properties [26]. Moreover, high-order modes in plates and pipes have been extensively studied for damage detection [27,28,29,30,31,32]. They can provide increased sensitivity to small defects due to their relatively short wavelength [32]. Belanger [29] exploited multiple high-order shear horizontal (SH) modes simultaneously with different cutoff frequency-thickness products for corrosion detection. High-order modes excited at a high frequency-thickness regime were used to detect cracks and pinholes in pipes [30]. They can even be employed for monitoring fatigue crack growth at fastener holes in metallic components [31]. However, the study of wave-damage interaction involving high-order modes is very limited at the present moment due to complicated phenomena. To facilitate the understanding of SHM applications using high-order modes, it is crucial to investigate the wave-damage interaction by extracting the WDIC of all the propagating wave modes. To the best of our knowledge, WDIC extraction of high-order modes in isotropic plates has not been reported.

The aim of this paper is to explore the effectiveness of employing modal decomposition to extract WDIC of high-order modes. The commercial finite element package ANSYS 17.0 was used to implement and realize the local FEM model for WDIC extraction of high-order modes in isotropic materials. First, the principle and procedure of WDIC extraction through modal decomposition were introduced. Then, the local FEM model was conducted. Finally, the case studies were carried out to validate the proposed method for high-order WDIC extraction of a through-thickness hole and a sub-surface crack in a 6-mm aluminum plate.

## 2. WDIC Extraction of High Order Modes through Modal Decomposition

### 2.1. The Use of WDIC in HGL Analysis

**Figure 1:**
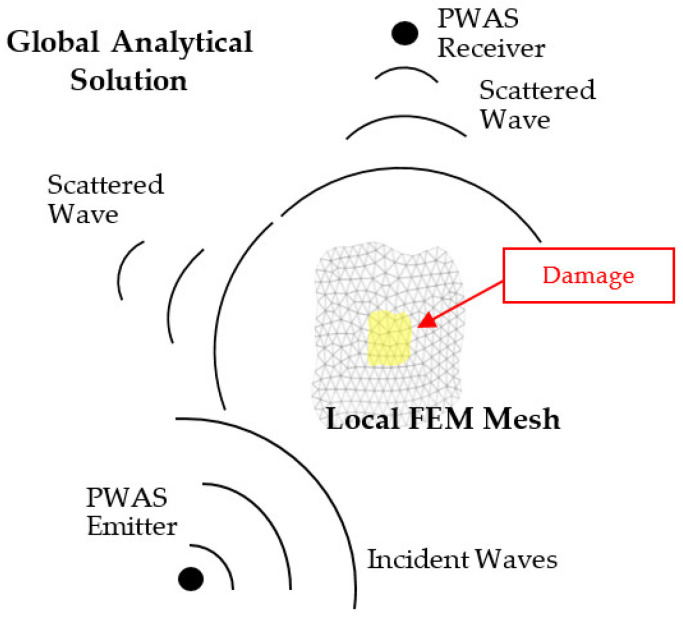
General two-dimensional (2D) setup for hybrid global-local modeling of structural sensing.

To address guided-wave SHM of aerospace structures, Mal and co-workers [33] developed a combination of closed-form analytical solution in the global domain and FEM solution in the local domain to achieve an efficient simulation of guided wave propagation and interaction with damage in thin plates. An HGL application to arbitrary waveguides using the semi-analytical finite element (SAFE) method is described in ref. [34]. In contrast with ref. [33], a different approach was adopted to combine frequency-domain and time-domain solutions and to use a transition overlap between the local and global regions [23]. In this approach, the guided waves generated by a PWAS transmitter were scattered from damage and were picked up by a PWAS receiver (Figure 1). The travel from the transmitter PWAS to the damage and from the damage to the receiver PWAS is modeled with analytical wave propagation formulae.

The approach to the HGL method was to replace the damage with a new wave source that generated the scattered wavefield to be added to the analytically calculated ‘pristine’ field. The scattered field was defined in terms of the complex-valued WDIC that were calculated separately [23]. The propagation of the scattered wavefield was also done analytically. The HGL methodology incorporated an analytical framework for guided wave propagation in the global region (called Wave Form Revealer) which allowed for the insertion of localized scatterers at user-defined locations to account for damage effects [35]. The damage scatterers were described as complex-numbered WDIC values, which were crucial to model wave-damage interaction in HGL analysis.

### 2.2. Modal Decomposition

**Figure 2:**
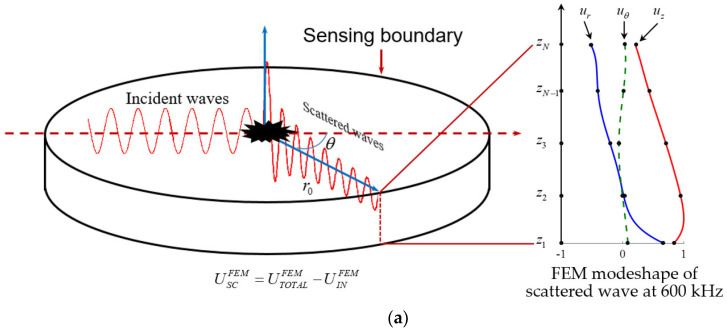

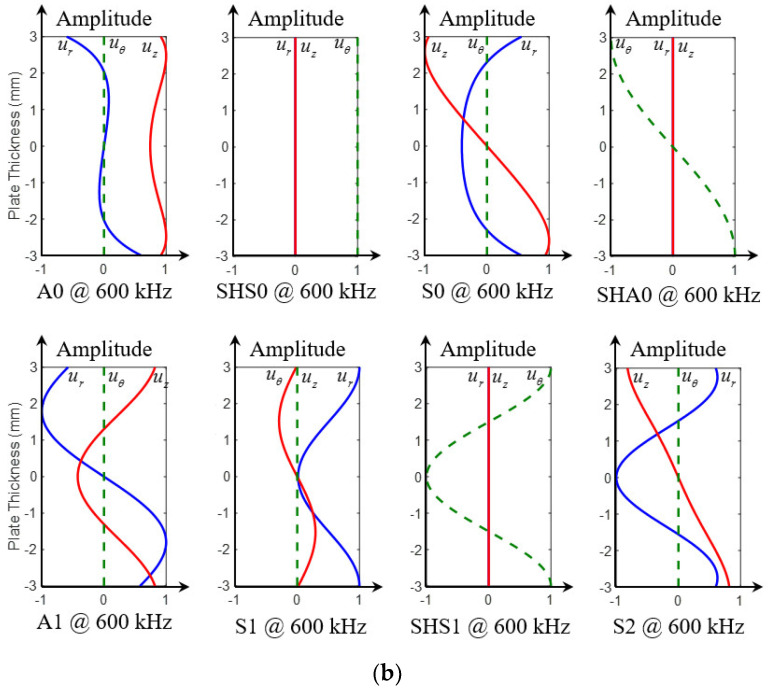
Schematic of wave-damage interaction coefficients (WDIC) extraction using modal decomposition: (**a**) typical finite element model (FEM) modeshape of scattered wave at 600 kHz; (**b**) analytical displacement modeshape of all eight wave modes in a 6-mm thick aluminum plate at 600 kHz.

Figure 2 shows the schematic of WDIC extraction for high-order modes using modal decomposition. In the analytical framework, the received signal was comprised of two parts: direct incident waves and scattered waves from damage. Thus, the damage could be modeled as a secondary wave source. The fundamental assumption in the proposed approach was that the incident wavefield and the scattered wavefield were additive because the medium had a linear elastodynamic behavior. When the incident wave interacted with the damage, the wave-damage interaction would generate a scattered wavefield that could be decomposed through projection on the inherent wave modes of the wave-guide medium (here, a plate). The decomposition process assumed simultaneous existence of all the wave modes used as a decomposition basis. The wave guide was two-dimensional (2D), hence propagation happening in all directions. In order to simulate the damage effect, the scattered wavefield due to damage was considered a new wave source which was linearly added to the incident wavefield. 

The total wavefield was the superposition of the incident wavefield and scattered wavefield from the damage. In order to extract the WDIC of high-order modes, the scattered wavefield had to be obtained. First, local FEM harmonic analysis was utilized to calculate the incident wavefield $U_{IN}^{FEM}$ in a pristine plate and total wavefield $U_{TOTAL}^{FEM}$ in a damaged plate. Thus, the scattered wavefield $U_{SC}^{FEM}$ from damage was determined, which was the difference between total wavefield from damaged model and incident wavefield from the pristine model, as given in Equation (1)

(1) $$U_{SC}^{FEM}=U_{TOTAL}^{FEM}-U_{IN}^{FEM}$$

A typical FEM modeshape of the scattered wave at 600 kHz in a 6-mm thick aluminum plate is presented in Figure 2a, *u_r_*, *u_θ_* and *u_z_* displacement components are plotted. These total displacement components picked up at the sensing boundary were the superposition of displacements from all the wave modes. It was found that they were neither symmetric nor antisymmetric with respect to the center of the plate thickness.

The displacement modeshapes of all eight wave modes at 600 kHz are given in Figure 2b, it can be observed that different modes have various modeshape characteristics. For example, symmetric Lamb wave modes (S0, S1 and S2) had symmetric *u_r_* component and antisymmetric *u_z_* component across the thickness, whereas antisymmetric *u_r_* component and symmetric *u_z_* component were observed for all antisymmetric Lamb wave modes (A0 and A1). All *u_θ_* components of Lamb wave modes were zero as expected [1]. On the other hand, shear horizontal modes only had non-zero *u_θ_* components. SHS0 and SHS1 modes had the symmetric *u_θ_* components whereas SHA1 mode had an antisymmetric *u_θ_* component. In order to find the WDIC of each mode, the modal decomposition had to be used to find the participation factors of each mode. The implementation of this approach is given in detail as follows.

To extract the WDIC information, scattered wave modes generated by the damage were assumed to propagate into the structure simultaneously. The outward propagating wavefield had been shown to follow Hankel function pattern [1]. The total displacement components were the superposition of displacements from all the wave modes. For example, $U_{r}^{FEM}\left(z\right)$ represented the *u_r_* displacement component from FEM solution at a thickness location *z* and consisted of the superposition of *u_r_* components contributed by all the wave modes. At discretized thickness-wise locations, this relationship is expressed as shown in Equation (2) below, i.e.,

(2) $$\left\{\begin{array}{c} \sum_{m=1}^{M}a_{m}\phi_{r}^{m}\left(z_{1}\;\right)H_{1}^{\left(1\right)}\left(\xi^{m}r_{0}\right)=U_{r}^{FEM}\left(z_{1}\right)\\ \sum_{m=1}^{M}a_{m}\phi_{r}^{m}\left(z_{2}\right)H_{1}^{\left(1\right)}\left(\xi^{m}r_{0}\right)=U_{r}^{FEM}\left(z_{2}\right)\\ \vdots \\ \sum_{m=1}^{M}a_{m}\phi_{r}^{m}\left(z_{N}\right)H_{1}^{\left(1\right)}\left(\xi^{m}r_{0}\right)=U_{r}^{FEM}\left(z_{N}\right) \end{array}\right.$$

where *a_m_* is the mode participation factor of the *m*^th^ wave mode, i.e., WDIC of each mode; $\phi_{r}^{m}(z_{n})$ is the analytical *u_r_* component of the *m*^th^ wave modeshape at location *z_n_*, *n* = 1, 2,…, *N*; $H_{1}^{\left(1\right)}$ is Hankel function of the first kind and order one describing an outward propagating wavefield; $\xi^{m}$ is the wavenumber of the *m*^th^ wave mode; *r*_0_ is the distance between the damage center and the sensing boundary. Similar relationships can be obtained for *u_θ_* and *u_z_* displacement components. These relationships are described using analytical expressions. The modeshapes and wavenumbers are calculated using the analytical solutions of the Rayleigh–Lamb equation [1]. The $U_{r}^{FEM}\left(z\right)$ are calculated from the local FEM. Thus, a series of linear equations with the mode participation factors as the only unknown quantities was obtained. Since the number of equations was greater than the number of unknowns and increased as the thickness direction mesh was further densified in the local FEM. Finally, an overdetermined equation system was obtained, which was solved with the least square method using MATLAB. The equation system is expressed in matrix form as

(3) $$\Phi HA=U_{SC}^{FEM}$$

where Φ is the modeshape matrix; **H** is the wave propagation matrix; **A** is the unknown mode participation factor vector (i.e., WDIC of each wave mode) to be solved. $U_{SC}^{FEM}$ is the FEM solution vector for scattered waves, which is the difference between the total wavefield and incident wavefield. These matrices and vectors are given as follows.

(4) $$\Phi =\left[\begin{array}{cccc} \phi_{r}^{1}\left(z_{1}\right) & \phi_{r}^{2}\left(z_{1}\right) & \cdots & \phi_{r}^{M}\left(z_{1}\right)\\ \vdots & & & \vdots \\ \phi_{r}^{1}\left(z_{N}\right) & \phi_{r}^{2}\left(z_{N}\right) & \cdots & \phi_{r}^{M}\left(z_{N}\right)\\ \phi_{\theta }^{1}\left(z_{1}\right) & \phi_{\theta }^{2}\left(z_{1}\right) & \cdots & \phi_{\theta }^{M}\left(z_{1}\right)\\ \vdots & & & \vdots \\ \phi_{\theta }^{1}\left(z_{N}\right) & \phi_{\theta }^{2}\left(z_{N}\right) & \cdots & \phi_{\theta }^{M}\left(z_{N}\right)\\ \phi_{z}^{1}\left(z_{1}\right) & \phi_{z}^{2}\left(z_{1}\right) & \cdots & \phi_{z}^{M}\left(z_{1}\right)\\ \vdots & \vdots & & \vdots \\ \phi_{z}^{1}\left(z_{N}\right) & \phi_{z}^{2}\left(z_{N}\right) & \cdots & \phi_{z}^{M}\left(z_{N}\right) \end{array}\right]$$

(5) $$H=\left[\begin{array}{cccc} H_{1}^{\left(1\right)}\left(\xi^{1}r_{0}\right) & 0 & \cdots & 0\\ 0 & H_{1}^{\left(1\right)}\left(\xi^{2}r_{0}\right) & 0 & \vdots \\ \vdots & 0 & \vdots & 0\\ 0 & \cdots & 0 & H_{1}^{\left(1\right)}\left(\xi^{M}r_{0}\right) \end{array}\right]$$

(6) $$U_{SC}^{FEM}={\left[U_{r}^{FEM}\left(z_{1}\right)\cdots U_{r}^{FEM}\left(z_{N}\right)\;U_{\theta }^{FEM}\left(z_{1}\right)\cdots U_{\theta }^{FEM}\left(z_{N}\right)\;U_{z}^{FEM}\left(z_{1}\right)\cdots U_{z}^{FEM}\left(z_{N}\right)\right]}^{T}$$

## 3. Case Studies

### 3.1. Local Finite Element Model

**Figure 3:**
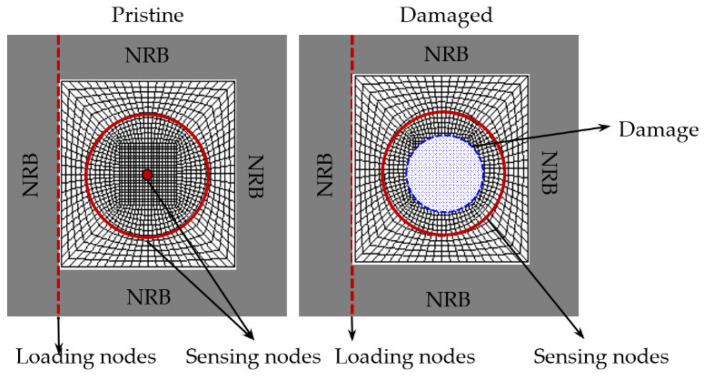
Schematic of local FEM pair for WDIC extraction.

In this section, the local FEM mesh was realized using commercial software ANSYS. Non-reflective boundaries (NRB) developed in ref. [36] could eliminate boundary reflections, and thus allow for simulation of guided wave propagation in an infinite medium. NRB was used in the local FEM harmonic analysis to simulate harmonic incident waves and scattered waves from wave-damage interaction. Two harmonic analyses, a pristine model and a damaged model, were conducted to extract WDIC for a certain incident wave mode. In this study, only fundamental S0 and A0 wave interaction with damage were considered. Thus, two pairs of harmonic analyses needed to be performed for a certain type of damage. The harmonic analysis was performed for any specific frequency of interest. The steady-state amplitude and phase information facilitated the extraction of WDIC. The schematic of local FEM pair designed for a 6-mm thick aluminum plate is given in Figure 3. The material properties are given in Table 1. Each local FEM model is 100 mm × 100 mm × 6 mm. A 30-mm wide NRB is applied to cover each boundary. COMBIN14 spring-damper elements were utilized to build the NRB. In this case, 3D solid structural elements (SOLID45) were employed to construct the aluminum plate.

The sensing nodes and loading nodes are shown in Figure 3. A sensing circle and one sensing node at the center of the pristine model were utilized to record response data. The sensing node at the center was used to collect the incident wave at the damage location for normalization. Only a sensing circle in the damaged model, with the same location as the pristine case, was used to record the structural response in harmonic analysis. In this study, only the propagating modes were considered which were picked up by the sensors placed in the global domain away from the damage, as appropriate for a guided-wave SHM system. The sensing circle for wave extraction in the FEM simulation was chosen to be large enough to avoid the near-field non-propagating modes generated from the damage during the wave-damage interaction. The design of the sensing circle was used to extract damage information for all directions. The sensing data in the pristine case corresponded to incident wavefield, while the sensing data from damaged case represented the total wavefield which was the superposition of scattered and incident waves. Therefore, the subtraction between pristine model and damage model provided the scattered wavefield in all directions. The loading nodes were used to simulate straight-crested incident wavefield, which was a good approximation when the damage is located far away from the excitation source. The wave mode excitation was imposed through nodal forces through boundary integration on each element along the loading line for each frequency.

The maximum acceptable element size to ensure convergence is *λ*_min_/*l_e_* ≥ 10, where *l_e_* is the element size and *λ*_min_ is the minimum wavelength [37]. The mesh size adopted in this study is 0.5 mm for in-plane direction and thickness direction since A0 mode has the minimum wavelength and it is around 5 mm. The damage regions were meshed with even smaller elements to guaranteed that more than 20 elements exist per wavelength, which was substantially more than the minimum requirement of 10 elements. It should be noted that different types of damage would have different scattering characteristics; this would require a corresponding local damage model for the WDIC extraction.

**Figure 4:**
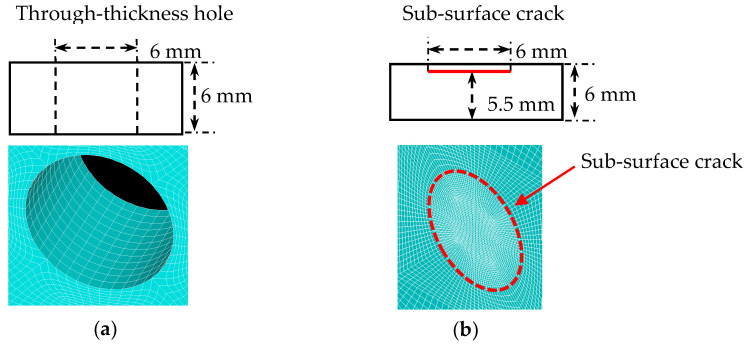
Damage type for WDIC extraction in 6-mm thick aluminum plate: (**a**) through-thickness hole; (**b**) sub-surface crack.

For the case studies, a 6-mm through-thickness hole and a circular sub-surface crack of 6-mm diameter were introduced in a 6-mm thick aluminum plate. Figure 4 shows the schematic of two damage types, a through-thickness hole and a sub-surface crack, designed for the 6-mm thick aluminum plate. The sub-surface crack was created at the depth of 0.5-mm from the top surface by specifying the crack as two surfaces, which were defined by the same coordinates but are not tied together. In this thick aluminum plate, high-order modes will exist at higher frequencies.

### 3.2. Dispersion Curves of 6-mm Thick Aluminum Plate

**Figure 5:**
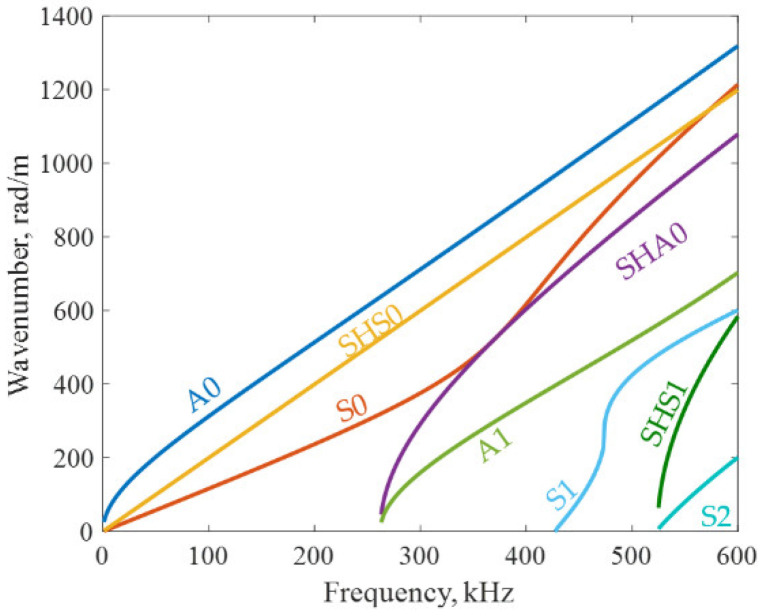
Frequency-wavenumber dispersion curves of a 6-mm thick aluminum plate.

In order to extract the WDIC of high-order modes, the frequency-wavenumber dispersion curves and displacement modeshapes had to be obtained. Figure 5 shows the frequency-wavenumber dispersion curves of the 6-mm thick aluminum plate. The frequency range is 0–600 kHz. It was found that there were only three fundamental modes, A0, SHS0 and S0, at low frequency (below 250 kHz). At 600 kHz, all eight wave modes (A0, SHS0, S0, SHA0, A1, S1, SHS1 and S2) existed.

**Figure 6:**
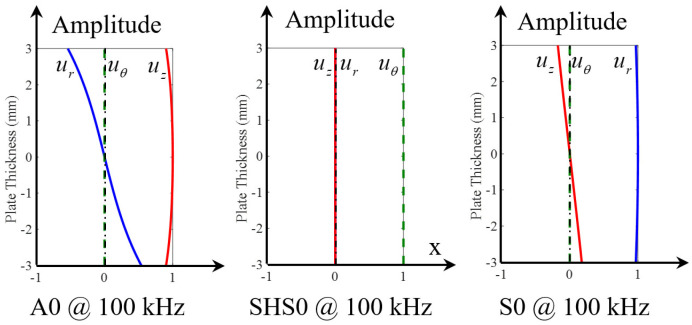
Displacement modeshapes at 100 kHz in a 6-mm thick aluminum plate showing three fundamental modes (A0, SHS0 and S0).

The displacement modeshapes of three fundamental modes at 100 kHz are given in Figure 6. For A0 mode, it can be noted that *u_r_* component is asymmetric across the thickness while *u_z_* component is symmetric across the thickness. On the contrary, S0 mode has a symmetric *u_r_* component and an asymmetric *u_z_* component across the thickness. *u_θ_* components of A0 and S0 modes are both zero. The only non-zero component of SHS0 is the symmetric *u_θ_* component.

**Figure 7:**
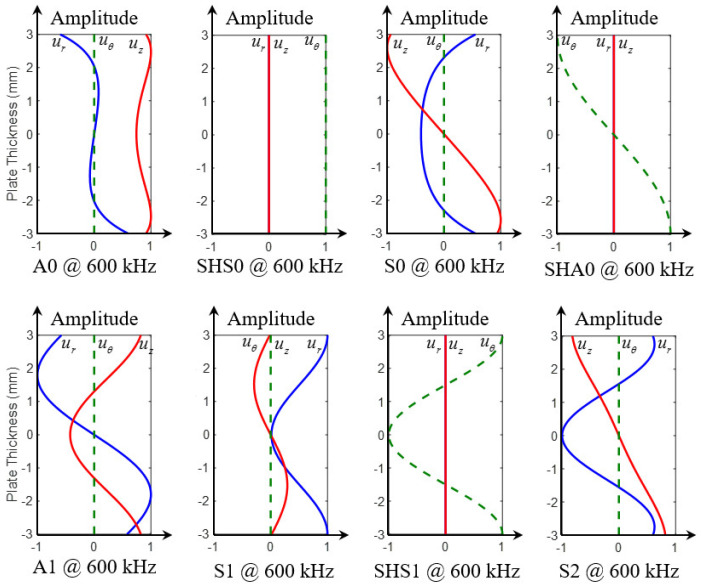
Displacement modeshapes at 600 kHz in a 6-mm thick aluminum plate showing all eight modes (fundamental modes: A0, SHS0 and S0; high-order modes: SHA0, A1, S1, SHS1 and S2).

Figure 7 shows the displacement modeshapes of all the wave modes at 600 kHz. It can be found that there are eight wave modes, three fundamental wave modes (A0, SHS0 and S0) and five high-order modes (SHA0, A1, S1, SHS1 and S2). Different wave modes have various modeshape characteristics. These displacement modeshapes and wavenumbers will be used to extract the WDIC of each mode through modal decomposition as discussed in Section 2.

### 3.3. WDIC of Through-Thickness Hole in a 6-mm Thick Aluminum Plate

#### 3.3.1. WDIC Verification

**Figure 8:**
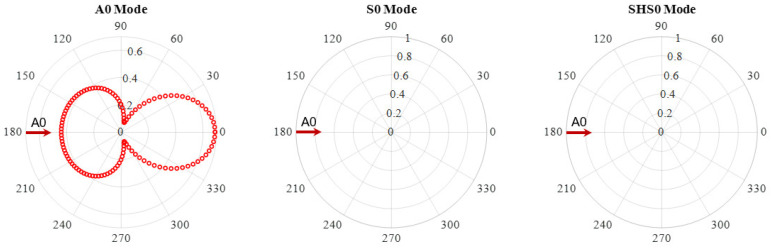
WDIC of the through-thickness hole at 100 kHz in 6-mm thick aluminum plate.

First, the WDIC extraction at 100 kHz was conducted, where only three fundamental modes (A0, S0 and SHS0) existed. Figure 8 shows an example of A0 wave interaction with the through-thickness hole in a 6-mm thick aluminum plate at 100 kHz. The amplitude coefficients of three wave modes were plotted. It was observed that the interaction between the incident A0 wave and the hole only involves scattered A0 wave, no mode conversion occurred for this symmetric through-thickness hole. This was consistent with results in ref. [38].

Another important feature is the direction dependence of the scattering amplitude. The amplitude coefficients are different for each scattering direction, i.e., they are heavily direction dependent. At 100 kHz, the scattered A0 possessed high amplitudes in 0° and 180° directions. In the case of a pitch-catch experiment, it is likely that the scattered A0 mode can be detected if the sensor is in the forward and backward directions of the incident wave. As shown in Figure 8, the scattering amplitudes around the directions perpendicular to the incident wave have relatively small amplitudes. Therefore, it is unlikely that the through-thickness can be detected if the sensor is located along these directions in a pitch-catch method.

**Figure 9:**
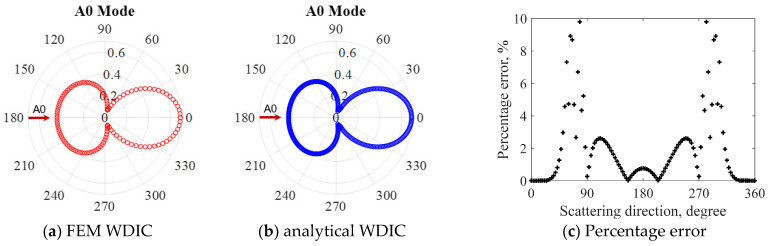
WDIC comparison of through-thickness hole between FEM results extracted through modal decomposition and analytical model for A0 incident at 100 kHz: (**a**) FEM WDIC; (**b**) Analytical WDIC; (**c**) Percentage error.

In order to verify the accuracy of the proposed WDIC extraction method, the comparison between FEM results and the analytical model for the through-thickness hole at 100 kHz was conducted, as shown in Figure 9. The analytical model of through-thickness hole for A0 incident can be found in ref. [38]. It was found that a good agreement between the two results was achieved, which demonstrated the accuracy of the proposed modal decomposition for WDIC extraction. Meanwhile, the percentage error *ε_θ_* defined in Equation (7) is used as a quantitative metric to quantify the accuracy of the proposed method.

(7) $$\varepsilon_{\theta }=\frac{\left|{\mathrm{WDIC}}_{FEM}^{\theta }-{\mathrm{WDIC}}_{analytical}^{\theta }\right|}{{\mathrm{WDIC}}_{analytical}^{\theta }}\times 100\%$$

where *θ* is the scattering direction, ${\mathrm{WDIC}}_{FEM}^{\theta }$ is the FEM WDIC at various scattering directions, ${\mathrm{WDIC}}_{analytical}^{\theta }$ is the analytical WDIC. Then percentage error between FEM results and the analytical model for the through-thickness hole at 100 kHz was calculated, as shown in Figure 9c. It was found that the maximum error was around 10% in 80° direction, where the minimum WDIC amplitude was observed. It should be noted that the error was less than 3% in the forward and backward scattering directions where the dominant WDIC was obtained.

#### 3.3.2. WDIC Extraction of High-Order Modes

**Figure 10:**
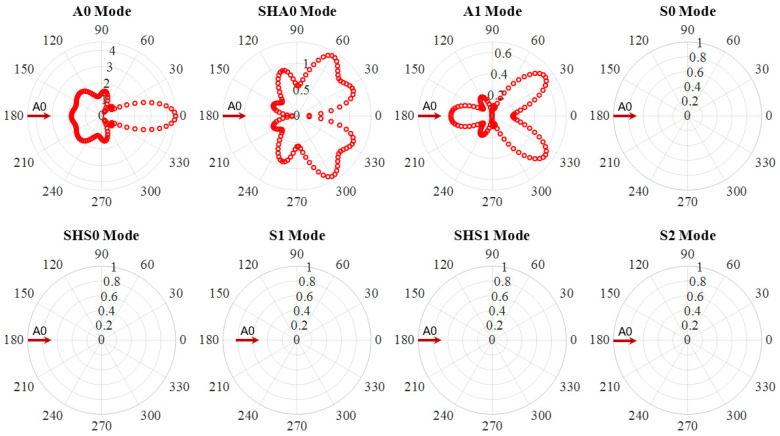
WDIC of through-thickness hole for A0 incident at 600 kHz in 6-mm thick aluminum plate showing mode conversion only happened between the antisymmetric modes (A0, SHA0 and A1), and the symmetric mode would not participate in the wave-damage interaction procedure.

In this section, the WDIC extraction of all wave modes at 600 kHz was carried out. Figure 10 shows an example of A0 wave interaction with through-thickness hole in a 6-mm thick aluminum plate at 600 kHz. The WDIC amplitude coefficients of all eight wave modes are plotted. It was observed that the A0 incident waves were scattered as A0 mode and converted to SHA0 and A1 mode. No symmetric wave modes were converted during the scattering procedure, i.e., when A0 wave interacted with the through-thickness hole, mode conversion only happened between the antisymmetric modes (A0, SHA0 and A1), and the symmetric mode would not participate in the wave-damage interaction procedure. This is because through-thickness hole was symmetric across the thickness. Another important feature was the direction dependence of the scattering amplitude. The amplitude coefficients are different for each scattering direction, i.e., they are heavily direction dependent. The WDIC of three wave modes were symmetric with respect to the 0° direction because the through-thickness hole was symmetric. For A0 incident, the dominant scattered wave was A0 mode through the WDIC amplitude comparison of all the antisymmetric modes (A0, SHA0 and A1). It was found that the scattered A0 mode had the largest amplitude in 0° direction. The mode converted A1 mode showed its highest forward amplitude in 40° and 320° directions. For mode converted SHA0 mode, it can be noted that there is no scattered wave in 0° and 180° directions. The maximum WDIC amplitudes are in 60° and 300° directions. Therefore, it is likely that the high-order mode converted waves can be detected if the sensor is in the forward directions of the incident wave where have the maximum WDIC amplitude.

**Figure 11:**
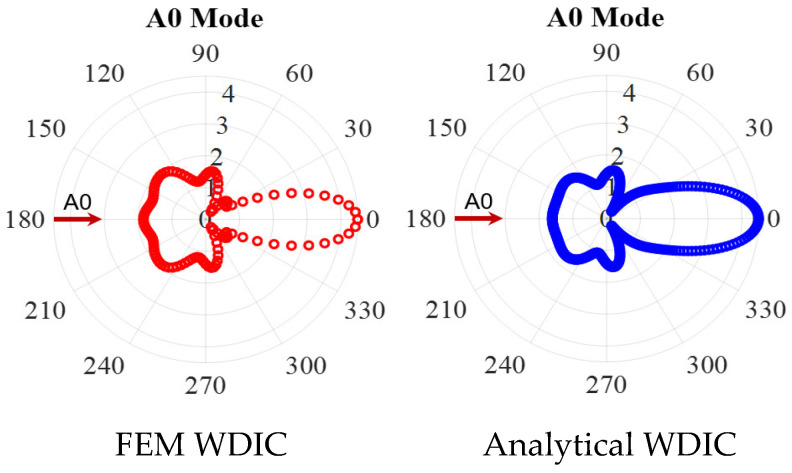
WDIC comparison of through-thickness hole between FEM results extracted through modal decomposition and analytical model for A0 incident at 600 kHz showing a good match between them.

To verify the accuracy of the proposed WDIC extraction method at high frequency where the convergence of the FEM model itself becomes more critical. The comparison between FEM results and the analytical model at 600 kHz was conducted, as shown in Figure 11. It was found that a good match between these two results was achieved, which demonstrated the accuracy of the proposed method for WDIC extraction at high frequency. It should be noted that a small deviation was observed between two WDIC pattern in the backward direction, this was because the analytical model was based on Mindlin plate theory, which was used to model flexural waves. However, it was found that the flexural waves were just a low-frequency approximation of the A0 Lamb wave mode [1]. Hence, the deviation between FEM and analytical model will increased as the frequency increased.

**Figure 12:**
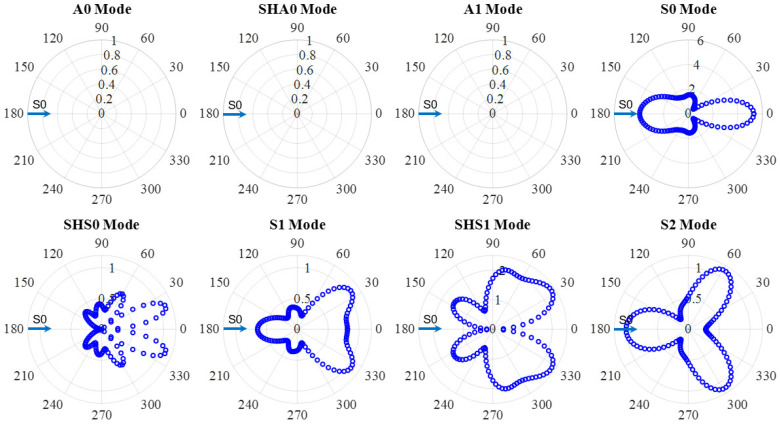
WDIC of through-thickness hole for S0 incident at 600 kHz in 6-mm thick aluminum plate showing mode conversion only happened between the symmetric modes (S0, SHS0, S1, SHS1 and S2), and the antisymmetric mode would not participate in the wave-damage interaction procedure.

Figure 12 shows an example of S0 wave interaction with through-thickness hole in a 6-mm thick aluminum plate at 600 kHz. The amplitude coefficients of all eight wave modes are plotted. It can be noted that the S0 incident waves were scattered as S0 mode and converted to SHS0, S1, SHS1 and S2 modes. Aside from the WDIC of fundamental S0 mode, the WDIC of high-order modes (S1, SHS1 and S2) were successfully extracted through modal decomposition. Similarly, no antisymmetric wave modes were converted during the scattering procedure, i.e., when S0 wave interacted with the through-thickness hole, mode conversion only happened between the symmetric modes (S0, SHS0, S1, SHS1 and S2), and the antisymmetric mode would not participate in the wave interaction with this symmetric through-thickness hole. For S0 incident, the dominant scattered wave was S0 mode which had the largest WDIC. Another important feature was the direction dependence of the scattering amplitude. The amplitude coefficients are different for each scattering direction, i.e., they are heavily direction dependent. The WDIC of five wave modes are symmetric with respect to the 0° direction because the through-thickness hole was symmetric. Scattered S0 mode has large WDIC amplitude in the forward and backward directions. High-order Lamb wave modes (S1 and S2) have a different WDIC pattern with more lobes. For high-order mode S2, it can be observed that there are three main lobes, two of them in the forward directions (60° and 300°) and one in the backward direction (180°). For shear horizontal modes, it can be noted that WDIC amplitudes are zero in 0° and 180° directions for SHS0 and SHS1 modes. In order to detect the scattered wave of high-order modes, it is important to put the sensor in the direction where has the highest WDIC.

For the WDIC of through-thickness hole for A0 and S0 incidents at 600 kHz, it was found that for symmetric wave incident, the mode conversion only happened between the symmetric modes, and the antisymmetric mode would not participate in the scattering procedure, vice versa. This was because the through-thickness hole was symmetric across the thickness.

### 3.4. WDIC of Sub-Surface Crack in a 6-mm Thick Aluminum Plate

**Figure 13:**
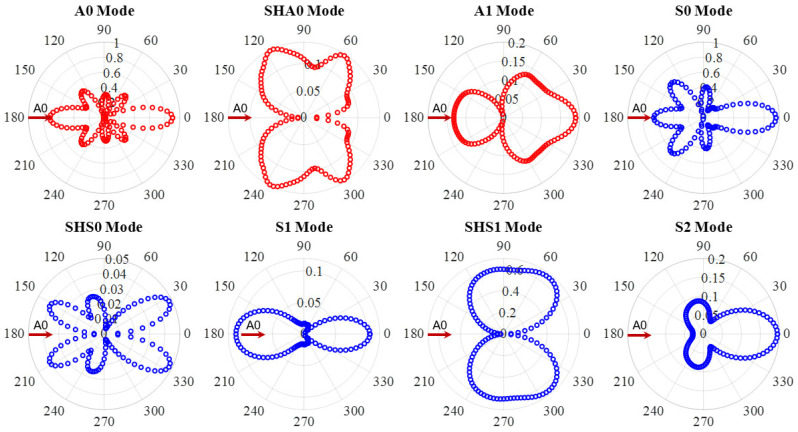
WDIC of sub-surface crack for A0 incident at 600 kHz in 6-mm thick aluminum plate showing mode conversion between symmetric modes and antisymmetric modes.

In this section, the WDIC extraction of an asymmetric sub-surface (Figure 4b) across the thickness in a 6-mm aluminum plate was conducted. Figure 13 shows an example of A0 wave interaction with a sub-surface crack at 600 kHz. The amplitude coefficients of all eight wave modes are plotted. It can be observed that the A0 incident waves were scattered as A0 mode and converted to antisymmetric modes (SHA0 and A1) and symmetric modes (S0, SHS0, S1, SHS1 and S2). In contrast with WDIC of through-thickness hole, antisymmetric modes, as well as symmetric modes, were converted during the scattering procedure for sub-surface crack, i.e., when A0 wave interacted with the asymmetric sub-surface crack, mode conversion not only happened between the antisymmetric modes (A0, SHA0 and A1) but also occurred between symmetric modes (S0, SHS0, S1, SHS1 and S2) in the wave-damage interaction procedure. This was because the sub-surface crack was asymmetric across the thickness. As shown in Figure 13, aside from the WDIC of fundamental modes, the WDIC of high-order wave modes (SHA0, A1, S1, SHS1 and S2) were successfully extracted through the proposed modal decomposition method. For A0 incident, the dominant scattered wave were the A0 and S0 modes which had the largest WDIC. Another important feature is the direction dependence of the scattering amplitude. The amplitude coefficients are different for each scattering direction, i.e., they are heavily direction dependent.

**Figure 14:**
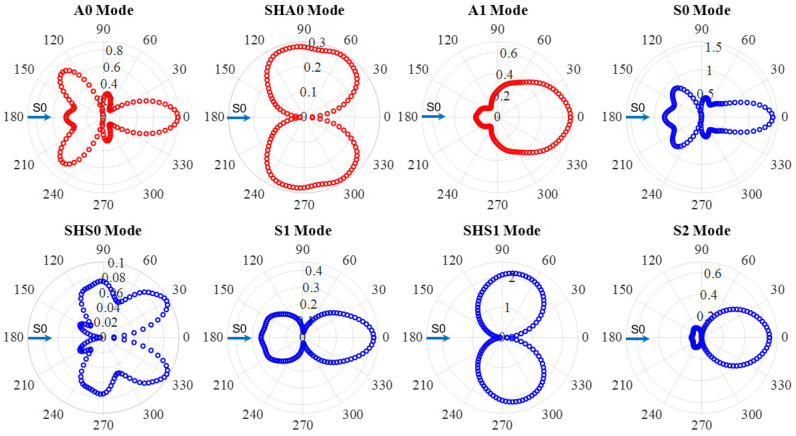
WDIC of sub-surface crack for S0 incident at 600 kHz in 6-mm thick aluminum plate showing mode conversion between symmetric modes and antisymmetric modes.

Figure 14 shows an example of S0 wave interaction with the sub-surface crack in a 6-mm thick aluminum plate at 600 kHz. The amplitude coefficients of all eight wave modes are plotted. It can be observed that the S0 incident waves were scattered as S0 mode and converted to symmetric modes (SHS0, S1, SHS1 and S2) and antisymmetric modes (A0, SHA0 and A1). Symmetric modes, as well as antisymmetric modes, were converted during the scattering procedure, i.e., when S0 wave interacts with the sub-surface crack, mode conversion not only happened between the symmetric modes (S0, SHS0, S1, SHS1 and S2) but also occurred between antisymmetric modes (A0, SHA0 and A1) in the wave-damage interaction procedure. Another important feature is the direction dependence of the scattering amplitude. The amplitude coefficients are different for each scattering direction, i.e., they are heavily direction dependent. For S0 incident, the dominant scattered wave is S0 and SHS1 modes which have the largest WDIC.

For the WDIC of sub-surface crack for A0 and S0 incidents at 600 kHz, it was found that the mode conversion happened between both the symmetric modes and the antisymmetric mode in the scattering procedure for either A0 or S0 incident. This is because the sub-surface crack is asymmetric across the thickness. WDIC of all the high-order modes were successfully extracted through proposed modal decomposition method.

## 4. Summary, Conclusions and Future Work

### 4.1. Summary

This paper explored the effectiveness of employing modal decomposition to extract WDIC of high-order modes. First, the framework of WDIC extraction through modal decomposition was introduced. Then, the local FEM model was conducted. The commercial finite element package was used to implement and realize the local FEM model for WDIC extraction of high-order modes in isotropic materials. Finally, two case studies were carried out to validate the proposed method for high-order WDIC extraction of a through-thickness hole and a sub-surface crack in a 6-mm thick aluminum plate. Analytical WDIC of through-thickness hole for A0 incident was utilized to verify the proposed approach. The proposed method can be used to analyze other flaws like local porosity and/or manufacturing defects. However, if the plate has a large area of pore or the size of manufacturing defects is too large, then the point-source assumption for the scatter waves may not be entirely true.

### 4.2. Conclusions

Aside from the WDIC of fundamental modes, the WDIC of high-order wave modes (SHA0, A1, S1, SHS1 and S2) were successfully extracted through the proposed modal decomposition method. The comparison between the FEM WDIC and the analytical WDIC of A0 wave scattering at through-thickness hole demonstrated the accuracy of the modal decomposition approach for WDIC extraction. In the case of symmetric through-thickness hole, mode conversion only happened between the antisymmetric modes (A0, SHA0 and A1) for A0 incident, and the symmetric mode would not participate in the wave-damage interaction procedure, a similar phenomenon was observed for S0 incident, mode conversion only happened between the symmetric modes (S0, SHS0, S1, SHS1 and S2) for S0 incident. In the case of asymmetric sub-surface crack across the thickness, mode conversion not only happened between the antisymmetric modes (A0, SHA0 and A1) but also involved symmetric modes (S0, SHS0, S1, SHS1 and S2) in the wave-damage interaction procedure for both A0 and S0 incidents. For the practical application, the proposed method will be used for predicting the performance of a structural health monitoring (SHM) system to be installed on structures. The WDIC pattern can be used to guide the sensor placement on the structures to receive a stronger scatted wave for better damage detection. Moreover, the WDIC information can be utilized in hybrid global local (HGL) approach to generate virtual data sets for testing data-driven models and for designing new SHM systems.

### 4.3. Future Work

An immediate extension of the current work would be in the WDIC extraction of high-order modes in composite plates. Experiments on thick specimens will be conducted to measure scattered waves of high-order mode to validate the proposed method. HGL approach will be used to predict the scattered waves of high-order modes using the extracted WDIC in SHM application for damage detection.

## Figures and Tables

**Table 1:** Material properties of aluminum 2024-T3 plate.

Young’s Modulus (E)	Poisson’s Ratio (ν)	Density (ρ)
73.1 GPa	0.33	2780 kg/m^3^

## Data Availability

Not applicable.

## References

[1] Giurgiutiu V. (2007). Structural Health Monitoring with Piezoelectric Wafer Active Sensors.

[2] Su Z., Ye L., Lu Y. (2006). Guided Lamb waves for identification of damage in composite structures: A review. J. Sound Vib..

[3] Mei H., Haider M.F., Joseph R., Migot A., Giurgiutiu V. (2019). Recent Advances in Piezoelectric Wafer Active Sensors for Structural Health Monitoring Applications. Sensors.

[4] Goossens S., Berghmans F., Khodaei Z.S., Lambinet F., Karachalios E., Saenz-Castillo D., Geernaert T. (2021). Practicalities of BVID detection on aerospace-grade CFRP materials with optical fibre sensors. Compos. Struct..

[5] Mei H., Giurgiutiu V. (2019). Guided wave excitation and propagation in damped composite plates. Struct. Health Monit..

[6] Harb M.S., Yuan F.G. (2017). Barely visible impact damage imaging using non-contact air-coupled transducer/laser Doppler vibrometer system. Struct. Health Monit..

[7] Yuan S., Ren Y., Qiu L., Mei H. (2016). A Multi-Response-Based Wireless Impact Monitoring Network for Aircraft Composite Structures. IEEE Trans. Ind. Electron..

[8] Shen Y., Giurgiutiu V. (2013). Predictive modeling of nonlinear wave propagation for structural health monitoring with piezoelectric wafer active sensors. J. Intell. Mater. Syst. Struct..

[9] Norris A.N., Vemula C. (1995). Scattering of flexural waves on thin plates. J. Sound Vib..

[10] Fromme P., Sayir M.B. (2002). Measurement of the scattering of a Lamb wave by a through hole in a plate. J. Acoust. Soc. Am..

[11] McKeon J.C.P., Hinders M.K. (1999). Lamb wave scattering from a through hole. J. Sound Vib..

[12] Vemula C., Norris A.N. (1997). Flexural wave propagation and scattering on thin plates using Mindlin theory. Wave Motion.

[13] Grahn T. (2003). Lamb wave scattering from a circular partly through-thickness hole in a plate. Wave Motion.

[14] Wang C.H., Chang F.-K. (2005). Scattering of plate waves by a cylindrical inhomogeneity. J. Sound Vib..

[15] Cegla F.B., Rohde A., Veidt M. (2008). Analytical prediction and experimental measurement for mode conversion and scattering of plate waves at non-symmetric circular blind holes in isotropic plates. Wave Motion.

[16] Moreau L., Caleap M., Velichko A., Wilcox P.D. (2012). Scattering of guided waves by flat-bottomed cavities with irregular shapes. Wave Motion.

[17] Velichko A., Wilcox P.D. (2010). A generalized approach for efficient finite element modeling of elastodynamic scattering in two and three dimensions. J. Acoust. Soc. Am..

[18] Fromme P. (2019). Guided wave sensitivity prediction for part and through-thickness crack-like defects. Struct. Health Monit..

[19] Veidt M., Ng C.T. (2011). Influence of stacking sequence on scattering characteristics of the fundamental anti-symmetric Lamb wave at through-thickness holes in composite laminates. J. Acoust. Soc. Am..

[20] Ng C.T., Veidt M. (2011). Scattering of the fundamental anti-symmetric Lamb wave at delaminations in composite laminates. J. Acoust. Soc. Am..

[21] Ng C.-T., Veidt M., Rose L.R.F., Wang C.H. (2012). Analytical and finite element prediction of Lamb wave scattering at delaminations in quasi-isotropic composite laminates. J. Sound Vib..

[22] Murat B.I.S., Khalili P., Fromme P. (2016). Scattering of guided waves at delaminations in composite plates. J. Acoust. Soc. Am..

[23] Shen Y., Giurgiutiu V. (2016). Combined analytical FEM approach for efficient simulation of Lamb wave damage detection. Ultrasonics.

[24] Bhuiyan M.Y., Shen Y., Giurgiutiu V. (2016). Guided wave-based crack detection in the rivet hole using global analytical with local FEM approach. Materials.

[25] Tian Z., Yu L. (2014). Lamb wave frequency–wavenumber analysis and decomposition. J. Intell. Mater. Syst. Struct..

[26] Bochud N., Laurent J., Bruno F., Royer D., Prada C. (2018). Towards real-time assessment of anisotropic plate properties using elastic guided waves. J. Acoust. Soc. Am..

[27] Dubuc B., Ebrahimkhanlou A., Salamone S. (2018). Higher order longitudinal guided wave modes in axially stressed seven-wire strands. Ultrasonics.

[28] Yu X., Zuo P., Xiao J., Fan Z. (2019). Detection of damage in welded joints using high order feature guided ultrasonic waves. Mech. Syst. Signal Process..

[29] Bélanger P. (2014). High order shear horizontal modes for minimum remnant thickness. Ultrasonics.

[30] Satyarnarayan L., Chandrasekaran J., Maxfield B., Balasubramaniam K. (2008). Circumferential higher order guided wave modes for the detection and sizing of cracks and pinholes in pipe support regions. NDT E Int..

[31] Masserey B., Fromme P. (2015). In-situ monitoring of fatigue crack growth using high frequency guided waves. NDT E Int..

[32] Ostachowicz W., Kudela P., Malinowski P., Wandowski T. (2009). Damage localisation in plate-like structures based on PZT sensors. Mech. Syst. Signal Process..

[33] Chang Z., Mal A.K., Ju J.W. (1995). A global-local method for wave propagation across a lap joint. Numerical Methods in Structural Mechanics.

[34] Srivastava A. (2009). Quantitative Structural Health Monitoring Using Ultrasonic Guided Waves. Ph.D. Thesis.

[35] Shen Y., Giurgiutiu V. (2014). WaveFormRevealer: An analytical framework and predictive tool for the simulation of multi-modal guided wave propagation and interaction with damage. Struct. Heal. Monit..

[36] Shen Y., Giurgiutiu V. (2015). Effective non-reflective boundary for Lamb waves: Theory, finite element implementation, and applications. Wave Motion.

[37] Moser F., Jacobs L.J., Qu J. (1999). Modeling elastic wave propagation in waveguides with the finite element method. NDT E Int..

[38] Mei H., Giurgiutiu V. Wave damage interaction in metals and composites. Proceedings of the SPIE Smart Structures + Nondestructive Evaluation.

